# Evolution of the Quality of Care in Patients with Decompensated Heart Failure in a Venezuelan Hospital

**DOI:** 10.3390/jcm14020644

**Published:** 2025-01-20

**Authors:** Yaneth Torres, Daniel Benitez, Zenaida Morillo, Juan Salazar, Julio Contreras-Velasquez, Valmore Bermudez

**Affiliations:** 1 Servicio de Cardiología, Hospital General del Sur, Maracaibo 4001, Venezuela; skylark21980@gmail.com (Y.T.); danieljosebenitezcamacho@gmail.com (D.B.); zenamorillo@hotmail.es (Z.M.); 2 Instituto de Investigaciones de Enfermedades Cardiovasculares de La Universidad del Zulia (IECLUZ), Maracaibo 4001, Venezuela; 3 Departamento de Productividad e Innovación, Universidad de la Costa, Barranquilla, Atlántico 080002, Colombia; jcontrer30@cuc.edu.co; 4 Universidad Simón Bolívar, Facultad de Ciencias de la Salud, Centro de Investigaciones en Ciencias de la Vida, Barranquilla 080001, Colombia

**Keywords:** quality of healthcare, heart failure, prognosis, follow-up, mortality

## Abstract

**Background/Objectives:** Several parameters have been proposed for the objective measurement of the quality of care (QoC) and breaches of care in patients with heart failure (HF). Therefore, the objective of this study was to evaluate the measures of QoC in inpatients with decompensated HF in the cardiology department of a tertiary Venezuelan hospital. **Methods**: An observational, descriptive, ambispective study was conducted with adults of all genders diagnosed with decompensated HF between 2022 and 2024. Sociodemographic, clinical, and therapeutic variables were assessed, as well as psychobiologic habits, measures of QoC, readmissions, and in-hospital mortality within the first 6 months of care. **Results**: Among the 131 subjects evaluated, the average age was 63.6 ± 14.1 years, with 58% (n = 76) being male. Among the in-hospital measures of QoC, the most common was the programming for follow-up consultations (98.5%; n = 129), followed by the prescription of beta-blockers (90.1%; n = 118). An upwards trend was also observed in the later years regarding the frequency of left ventricle ejection fraction (LVEF) assessment (*p* < 0.001), and the use of iSGLT2 (*p* = 0.03). During follow-up, 36.6% of the patients died within 6 months, with those in NYHA class IV showing a higher probability of death (OR: 3.84; CI95%: 0.89–16.55; 0.04). **Conclusions**: The in-hospital measures for QoC in this population were similar to those in previous reports, with LVEF assessment and iSGLT2 prescription showing a particularly significant increase in recent years.

## 1. Introduction

Heart failure (HF) is a major public health problem in several regions across the world, as it is the final stage of all known chronic heart diseases, and translates to increasing incidence, prevalence, and healthcare-related costs. According to the latest report by the Heart Failure Society of America, roughly 6.7 million people live with HF in the American continent, and this prevalence is estimated to increase to 8.7 million for 2030, and 11.4 million for 2050 [1], while in Europe, data from the HFA Atlas showed an overall HF prevalence of 17 cases per 1000 people, with considerable heterogeneity across the countries [2], and in the four world regions of GBD 2019, there were 31.89 million prevalent cases of HF in Asia (including Asia and Oceania), with an age-standardized rate of 722.45 per 100,000 population, a figure lower than that in America (810.42 per 100,000 population) and Africa (709.89 per 100,000 population), and higher than that in Europe (606.61 per 100,000 population) [3]. There are no large-scale data regarding the epidemiology of HF in Venezuela [4].

In addition to its large incidence, hospitalization rates, and healthcare costs, the in-hospital mortality of HF has remained high in recent decades. This is probably because decompensations are common in advanced HF, with a high rate of readmissions, prolonged hospital stays, and other adverse outcomes tightly related to the quality of in-hospital care [5]. In this regard, the perception of the quality of care (QoC) with respect to healthcare services is very subjective and hinges on the perspectives of each person. The patient wishes to receive integral care that covers their needs, while the healthcare personnel seek to offer optimal care within the parameters established by each institution. Lastly, health institutions look to articulate interdisciplinary teams to deliver the best care. In summary, the dimensions for measuring the quality of healthcare services center on scientific technical quality, user satisfaction, and service accessibility [6,7].

However, in regard to HF, several parameters have been proposed to objectively appraise QoC or breaches in the care of this group of patients, based on current recommendations by diagnostic and treatment guides for this disease. These have been recently updated given the important advances in therapeutics—especially concerning HF with reduced ejection fraction—and they contemplate the basic standards for appropriate care [8,9,10]. Given the scarcity of epidemiologic data on patients with HF and their in-hospital care in Venezuela, our hospital has begun research on the diagnostic and therapeutic approach to this population in our region. In this context, this is an initial exploratory study whose objective is to evaluate the measures of quality of clinical care in inpatients with decompensated HF in the cardiology department of a tertiary Venezuelan hospital.

## 2. Materials and Methods

### 2.1. Study Design and Sample Selection

An observational, descriptive, ambispective study was conducted on adults of all genders diagnosed with decompensated HF admitted to the Department of Cardiology of the General Hospital of the South “Dr. Pedro Iturbe” of the Zulia State within the period from June 2022 to April 2024. The institutional ethics committee approved the investigation under code HGS-2022, and all the analysis was conducted under the Declaration of Helsinki. Sample selection was performed from clinical reports from the department of medical data of this hospital, as per the following inclusion criteria: subjects of all genders, 18 years of age or older, admitted for the first time at the hospital with the diagnosis of decompensated HF, according to the corresponding guidelines for the diagnosis and treatment by the European Society of Cardiology [8]. The following were exclusion criteria: patients with incomplete clinical reports, those who had a previous hospital admission, those who were hemodynamically unstable and required admission into the intensive care unit, those with a hospital stay under 24 h, those who died within the first 24 h in the hospital, those who left the hospital against medical advice, those with mental disorders or other conditions which could prevent them from answering questions related to this study, and those who refused to participate in the study.

### 2.2. Patient Evaluation

A questionnaire was applied to obtain information from the medical reports, which were obtained within the first 24–48 h of the patient’s hospital stay. The following data were extracted: gender (male/female), age, marital status (single/married/widowed/other), educational status (analphabet/basic/secondary/higher), occupational status (unemployed/employed/retired), psychobiologic habits (smoking habits, drinking habits, regular exercise), cause of HF (ischemic/non-ischemic), NYHA classification (II/III/IV), and number of hospitalizations in the previous 12 months. The drugs used were also recorded: angiotensin-converting-enzyme inhibitors/angiotensin receptor blockers (ACEIs/ARBs), beta-blockers (BBs), mineralocorticoid receptor antagonists (MRAs), angiotensin receptor-neprilysin inhibitors (ARNIs), and Sodium-glucose cotransporter-2 inhibitors (SGLT2i); comorbidities were also recorded: hypertension, diabetes mellitus, obesity, anemia, high LDL-C, coronary artery disease (CAD), and chronic kidney disease (CKD).

Subsequently, the measures of in-hospital QoC proposed by Gupta et al. [9] were assessed, including the evaluation of left ventricle ejection fraction (LVEF) during hospitalization, the programming of a follow-up consultation after discharge, and the prescription of BBs (carvedilol, bisoprolol, or metoprolol succinate) and ACEIs/ARBs before discharge. However, upon the update in the standards of QoC published by the AHA/ACC in 2024 [10], information about other disease-modifying drugs was also obtained, specifically, whether MRAs or SGLT2i were prescribed before discharge.

Finally, as the database was created, the subjects registered were contacted by telephone by two researchers at 6 months after their date of discharge, in order to investigate the number of hospital readmissions or death by any cause during this period.

Given certain limitations for the application of questions related to QoC in our population, the questionnaire underwent the following preparative steps:

1. Direct and inverse translation by two independent translators unrelated to the study, one of them linked to the healthcare area.
2. Review and consolidation by a committee of experts from our institution.
3. Pre-test evaluation to assess viability in a pilot control group of 10 patients, who were excluded from the final analysis.

### 2.3. Statistical Analysis

The results were expressed as mean ± SD for quantitative variables, and as absolute numbers and percentages for qualitative variables. The Chi-squared test was utilized to evaluate the association between categorical variables. A binary logistic regression model was constructed for the prediction of all-cause mortality at 6 months of follow-up after discharge; this model included all variables with statistically significant results in the univariate analysis: year of admission, age group, marital status, NYHA classification, presence of anemia, use of SGLT2i before admission, and readmissions before 6 months. All analyses were conducted with the statistical software SPSS v25 for Windows (Chicago, IL, USA), and the alpha level was fixed at 0.05.

## 3. Results

Among the 131 subjects evaluated, the overall age was 63.6 ± 14.1 years, with 58% (n = 76) being males; 50.4% (n = 66) were <65 years of age, 48.1% (n = 63) were married, 34,4% (n = 45) had a basic or secondary educational level, and 49.6% (n = 20) were unemployed. The distribution of subjects evaluated per year of admission was 31.3% (n = 41) for 2022, 38.2% (n = 50) for 2023, and 30.5% (n = 40) for 2024. The general characteristics of the sample according to the year of admission are shown in Table 1.

In regard to psychobiologic habits, 40.5% (n = 53) of the subjects were smokers, 42.7% (n = 56) consumed alcohol regularly, and 98.5% (n = 129) were sedentary. Concerning comorbidities, hypertension (80.9%; n = 106), CAD (61.8%; n = 81), and CKD (38.2%; n = 50) were the most frequently reported. The distribution of subjects according to these features and their year of admission is shown in Table 2.

Table 3 summarizes the clinical and therapeutic characteristics of the subjects according to their year of admission. Notable features were the predominance of ischemic causes for HF (61.8%; n = 80), and the NYHA functional class IV (42.7%; n = 56). On the other hand, ACEIs/ARBs (61.8%; n = 80), BBs (55%; n = 72) and MRAs (33.6%; n = 44) were the main pharmacological groups used in this sample. Only 1.5% had a cardiac device. Among the in-hospital QoC measures assessed, the most frequent was the programming for a follow-up consultation (98.5%; n = 129), followed by the prescription of BBs (90.1%; n = 118) and LVEF evaluation (67.9%; n = 89). A growing trend was observed in the frequency of LVEF evaluation (*p* < 0.001) and SGLT2i use (*p* = 0.03) throughout the years (Table 4).

With respect to follow-up variables, the average of readmissions at 6 months was 2 ± 1, occurring in up to 25% (n = 33) of all subjects. Meanwhile, mortality was estimated at 36.6% (n = 48); with an ascending trend in the latter 2 years of study (*p* = 0.003) (Table 5). Among the factors associated with mortality, only subjects in NYHA class IV displayed a greater probability of death (OR: 3.84; CI95%: 0.89–16.55; 0.04) (Table 6).

## 4. Discussion

In current medical practice, clinical guidelines are purported as an instrument to facilitate physicians’ daily decision making, seeking to optimize the use of diagnostic tests and unify therapeutic approaches. The objective is to improve the patients’ QoC, always based on the scientific evidence available. In the case of HF, specific goals include the reduction in hospital admissions, avoiding unnecessary tests and treatments, and promoting the rational use of resources, and as a result, decreasing healthcare-related costs as well as morbidity and mortality in these patients, especially in low-income countries. This study depicts the profile of in-hospital QoC measure implementation over the past three years in a group of patients with decompensated HF admitted to a tertiary hospital from Maracaibo city, Venezuela.

The programming of early follow-up consultations, and the use of BBs, ACEIs/ARBs, and ARNIs before discharge were the QoC measures most frequently employed in our population. This differs from the observations by Gupta et al. [11] who in a sample of 10,000 admissions found the most common measures to be the in-hospital LVEF assessment, with 66.7% (45.5–80.7%), and the use of ACEIs or ARBs in patients eligible for discharge at 57.1% (36.4–75.0%). On the other hand, Yu et al. [12] ascertained substantial breaches of care in a Chinese population, including the underutilization of diagnostic tests such as echocardiography (63.6%), thoracic imaging (75.2%), and biomarkers (56.4%). Moreover, they also reported a low rate of prescription of guideline-recommended medication during hospitalization (67.7%) and even after discharge, suboptimal rates of follow-up consultations (24.3%), and the widespread utilization of traditional Chinese medicine (74.8%). These findings demonstrate a large heterogeneity in the management of a very frequent disorder worldwide, reflecting the influence of local economic, social, and cultural factors [13,14].

Notably, in our study, LVEF evaluation and SGLT2i prescription before discharge were the QoC measures that showed a rise in their utilization in the latter years. This is probably due to the increased availability of the material and human resources for performing echocardiography on all admitted patients; the improved knowledge of the prescription guidelines for SGLT2i and the importance of their use in most contexts of patients with HF; and the hard work implemented by the pharmaceutical industry to improve the availability of these drugs in our country, as has been illustrated in previous studies globally [15] and in our population [16]. However, it is still important to address certain barriers that influence its prescription such as cost, prescription in certain groups such as the elderly, patient perception, and preference, for which not only educational measures are needed but also support for the health system.

The lack of adherence to QoC measures may significantly increase readmission and mortality rates in the short- and mid-term evolution of heart failure patients [17,18]. Both of these outcomes were highly frequent in our population, even showing an increase in mortality in the past two years. Our findings in this regard are more severe than those reported by Tárraga et al. [19], who evidenced 16% mortality in an analysis of QoC in HF management in a basic health zone (Zona Básica de Salud) in Spain. Likewise, our mortality figures were also higher than those reported by He et al. [20] in a retrospective analysis on secondary and tertiary Chinese hospitals, where the mortality rates were 11% at 90 days after discharge and 21% after 1 year. This information denotes the need to emphasize the systematic compliance with the pharmacological QoC measures prior to discharge. Furthermore, it highlights the importance of evaluating other measures such as effective decongestion or the presence of other life-threatening comorbidities that may also influence these adverse outcomes and may not have been included in this study, and whose implementation may reduce the rates of readmission or mortality [21,22].

In this respect, although we identified some variables associated with mortality, such as marital status, the presence of anemia, and the non-use of SGLT2i, only subjects in NYHA class IV before admission exhibited a greater risk of death in the multivariate analysis. This underlines the importance of this simple tool in daily practice [23]. In relation to clinical and therapeutic characteristics before admission, it is important to note the low use of drugs such as MRAs, ARNIs, and SGLT2i, as well as a decrease in the percentage of use of ACEIs/ARBs and BBs in the past two years. This may be attributed to the socioeconomic difficulties our country is currently undergoing, which may be reflected in the acquisition of the medication and thus compliance with pharmacologic treatment [24]. Moreover, our findings underscore the importance of follow-up consultations (which are scheduled over long periods), in order to emphasize the need to continue disease-modifying therapies even in periods of clinical stability, and the promotion of the creation of support networks involving governmental and non-governmental entities, in order to guarantee access to these drugs and to improve access to device therapy [25].

Lastly, concerning comorbidities, the most common were hypertension, CAD, and cardiac arrhythmias. This diverts from the observations of Quian et al. [26] in an analysis of Asian American subjects without insurance or covered by Medicaid, who were shown to have a higher prevalence of diabetes, hypertension, and CKD than Caucasian subjects with similar LVEF. Both groups also had comparable QoC, with the exception of lower MRA prescription before discharge (RR: 0.88; CI95%: 0.78–0.99), and lower anticoagulation for atrial fibrillation (RR: 0.91; CI95%: 0.85–0.97) for the Asian American population, even after adjustment for risk. The notoriously high percentages of alcohol consumption and smoking, as well as low regular physical activity, in our population should be highlighted, since they are very important non-pharmacological risk factors for the management of these patients, and their optimization should be approached intensively after a hospital admission. This emphasizes the need to develop cardiovascular rehabilitation facilities in our region, in order to routinely manage these frequently underestimated aspects [27].

The limitations of this study include its design; since data were obtained from secondary sources, gaps in documentation may occur, possibly missing out some registries, especially regarding the behavior of high blood pressure or atrial fibrillation as a trigger for decompensation, which is currently being analyzed in another of our lines of research. Likewise, several types of QoC variables were not assessed, including those related to the hospital environment, specific socioeconomic or cultural factors, the use of multimodal imaging techniques such as MRI, and therefore the lack of determination of the causes of non-ischemic origin HF, and biologic factors such as natriuretic peptide and treatment compliance. These variables may also influence mortality and should be explored in further analyses.

## 5. Conclusions

The in-hospital QoC measures applied in the patients evaluated are similar to those reported in previous research, with programming for follow-up consultations and BB use being the most frequent, and LVEF assessment and SGLT2i prescription before discharge showing a significant increase in implementation in the more recent years. However, the percentages of readmission and mortality at 6 months are high in our population, with NYHA class IV being the main associated factor. In light of these findings, it is necessary to routinely evaluate QoC measures in inpatients with HF, appraising the compliance of the key clinical recommendations for this disease. Likewise, an essential point for further research is the assessment of potential additional factors that may impact the fulfillment of these QoC measures and the high mortality of our population.

## Figures and Tables

**Table 1:** General characteristics of the sample according to the year of admission.

	All (n = 131)		2022 (n = 41)		2023 (n = 50)		2024 (n = 40)		
	n	%	n	%	n	%	n	%	*p* *
Gender									0.70
Female	55	42%	16	39.0%	20	40.0%	19	47.5%	
Male	76	58%	25	61.0%	30	60.0%	21	52.5%	
Age group									0.99
<65 years	66	50.4%	21	51.2%	25	50.0%	20	50.0%	
≥65 years	65	49.6%	20	48.8%	25	50.0%	20	50.0%	
Marital status									0.003
Single	17	13%	7	17.1%	5	10.0%	5	12.5%	
Married	63	48.1%	27	65.9%	22	44.0%	14	35.0%	
Widowed	24	18.3%	7	17.1%	11	22.0%	6	15.0%	
Other	27	20.6%	0	0.0%	12	24.0%	15	37.5%	
Educational status									0.002
Analphabet	21	16%	10	24.4%	8	16.0%	3	7.5%	
Basic education	45	34.4%	12	29.3%	24	48.0%	9	22.5%	
Secondary education	45	34.4%	9	22.0%	13	26.0%	23	57.5%	
Higher education	20	15.2%	10	24.4%	5	10.0%	5	12.5%	
Occupational status									0.001
Unemployed	65	49.6%	9	22.0%	30	60.0%	26	65.0%	
Employed	43	32.8%	20	48.8%	13	26.0%	10	25.0%	
Retired	23	17.6%	12	29.3%	7	14.0%	4	10.0%	
Age (years)	63.6 ± 14.1		61.4 ± 15.3		65.5 ± 13.2		63.6 ± 14		0.38

* Chi-squared test.

**Table 2:** Psychobiologic habits and comorbidities of the sample according to the year of admission.

	All (n = 131)		2022 (n = 41)		2023 (n = 50)		2024 (n = 40)		
	n	%	n	%	n	%	n	%	*p* *
Smoking									0.004
No	78	59.5%	23	56.1%	23	46.0%	32	80.0%	
Yes	53	40.5%	18	43.9%	27	54.0%	8	20.0%	
Alcohol consumption									0.06
No	75	57.3%	29	70.7%	23	46.0%	23	57.5%	
Yes	56	42.7%	12	29.3%	27	54.0%	17	42.5%	
Regular exercise									0.11
No	129	98.5%	39	95.1%	50	100.0%	40	100.0%	
Yes	2	1.5%	2	4.9%	0	0.0%	0	0.0%	
Hypertension									0.26
No	25	19.1%	6	14.6%	8	16.0%	11	27.5%	
Yes	106	80.9%	35	85.4%	42	84.0%	29	72.5%	
Diabetes mellitus									0.36
No	88	67.2%	24	58.5%	36	72.0%	28	70.0%	
Yes	43	32.8%	17	41.5%	14	28.0%	12	30.0%	
Obesity									0.01
No	103	78.6%	30	73.2%	46	92.0%	27	67.5%	
Yes	28	21.4%	11	26.8%	4	8.0%	13	32.5%	
Anemia									0.001
No	90	68.7%	37	90.2%	28	56.0%	25	62.5%	
Yes	41	31.3%	4	9.8%	22	44.0%	15	37.5%	
High LDL-C									0.005
No	112	85.5%	29	70.7%	47	94.0%	36	90.0%	
Yes	19	14.5%	12	29.3%	3	6.0%	4	10.0%	
Cardiac arrhythmia									0.10
No	85	64.9%	24	58.5%	29	58%	32	80.0%	
Yes	46	35.1%	17	41.5%	21	42%	8	20.0%	
Coronary artery disease									0.15
No	50	38.2%	15	36.6%	15	30.0%	20	50.0%	
Yes	81	61.8%	26	63.4%	35	70.0%	20	50.0%	
Chronic kidney disease									0.34
No	81	61.8%	29	70.7%	28	56.0%	24	60.0%	
Yes	50	38.2%	12	29.3%	22	44.0%	16	40.0%	
Obstructive sleep apnea									0.96
No	129	98.5%	40	97.6%	50	100.0%	39	97.5%	
Yes	2	1.5%	1	2.4%	0	0.0%	1	2.5%	

* Chi-squared test.

**Table 3:** Clinical and therapeutic characteristics of the sample according to the year of admission.

	All (n = 131)		2022 (n = 41)		2023 (n = 50)		2024 (n = 40)		
	n	%	n	%	n	%	n	%	*p* *
Cause of heart failure									0.09
Ischemic	80	61.8%	26	63.4%	35	70.0%	19	47.5%	
Non-ischemic	51	38.2%	15	36.6%	15	30.0%	21	52.5%	
NYHA class									<0.001
II	27	20.6%	19	46.3%	8	16.0%	0	0.0%	
III	48	36.6%	17	41.5%	20	40.0%	11	27.5%	
IV	56	42.7%	5	12.2%	22	44.0%	29	72.5%	
ACEI/ARB use									0.02
No	51	38.2%	9	22.0%	25	50.0%	17	42.5%	
Yes	80	61.8%	32	78.0%	25	50.0%	23	57.5%	
Beta-blocker use									0.02
No	59	45%	11	26.8%	27	54.0%	21	52.5%	
Yes	72	55%	30	73.2%	23	46.0%	19	47.5%	
MRA use									0.37
No	87	66.4%	25	61.0%	32	64.0%	30	75.0%	
Yes	44	33.6%	16	39.0%	18	36.0%	10	25.0%	
ARNI use									0.51
No	119	78.6%	37	90.2%	44	88.0%	38	95.0%	
Yes	12	21.4%	4	9.8%	6	12.0%	2	5.0%	
SGLT2i use									0.25
No	100	76.3%	30	73.2%	42	84.0%	28	70.0%	
Yes	31	23.7%	11	26.8%	8	16.0%	12	30.0%	
ICD/CRT use									0.55
No	129	98.5%	41	100%	50	100%	38	95.0%	
Yes	2	1.5%	0	0%	0	0%	2	5.0%	
Hospitalizations in the preceding 12 months	1 ± 1		1 ± 1		1 ± 1		1 ± 1		0.97

* Chi-squared test. ACEI/ARB: Angiotensin-converting-enzyme inhibitor/angiotensin receptor blocker; MRA: Mineralocorticoid receptor antagonist; ARNI: Angiotensin receptor-neprilysin inhibitor; CRT: Cardiac resynchronization therapy; ICD: Implantable cardioverter defibrillator; SGLT2i: Sodium-glucose cotransporter-2 inhibitors.

**Table 4:** Quality of care measures applied during hospitalization on the evaluated subjects according to the year of admission.

	All (n = 131)		2022 (n = 41)		2023 (n = 50)		2024 (n = 40)		
	n	%	n	%	n	%	n	%	*p* *
LVEF evaluation									<0.001
No	42	32.1%	13	31.7%	29	58.0%	0	0.0%	
Yes	89	67.9%	28	68.3%	21	42.0%	40	100.0%	
Programming of follow-up consultation									0.62
No	2	1.5%	0	0.0%	1	2.0%	1	2.5%	
Yes	129	98.5%	41	100.0%	49	98.0%	39	97.5%	
Prescription of beta-blockers									0.02
No	13	9.9%	0	0.0%	9	18.0%	4	10.0%	
Yes	118	90.1%	41	100.0%	41	82.0%	36	90.0%	
Prescription of ACEIs/ARBs/ARNIs									0.07
No	24	18.3%	3	7.3%	13	26.0%	8	20.0%	
Yes	107	81.7%	38	92.7%	37	74.0%	32	80.0%	
Prescription of MRAs									0.99
No	50	38.2%	16	39.0%	19	38.0%	15	37.5%	
Yes	81	61.8%	25	61.0%	31	62.0%	25	62.5%	
Prescription of SGLT2i									0.03
No	72	55%	30	73.2%	23	46.0%	19	47.5%	
Yes	59	45%	11	26.8%	27	54.0%	21	52.5%	

* Chi-squared test. LVEF: Left ventricle ejection fraction; ACEIs/ARBs: Angiotensin-converting-enzyme inhibitors/angiotensin receptor blockers; MRAs: Mineralocorticoid receptor antagonists; ARNIs: Angiotensin receptor-neprilysin inhibitors; SGLT2i: Sodium-glucose cotransporter-2 inhibitors.

**Table 5:** Hospital readmissions and mortality at 6 months of follow-up on the evaluated subjects according to the year of admission.

	All (n = 131)		2022 (n = 41)		2023 (n = 50)		2024 (n = 40)		
	n	%	n	%	n	%	n	%	*p* *
Hospital readmissions									0.34
No	98	74.8%	34	82.9%	35	70.0%	29	72.5%	
Yes	33	25.2%	7	17.1%	15	30.0%	11	27.5%	
Mortality									0.003
No	83	63.4%	34	82.9%	24	48.0%	25	62.5%	
Yes	48	36.6%	7	17.1%	26	52.0%	15	37.5%	
Number of readmissions	2 ± 1		1 ± 1		2 ± 1		2 ± 1		0.11

* Chi-squared test.

**Table 6:** Factors associated with mortality on the evaluated subjects.

	Alive (n = 50)		Deceased (n = 40)			
	n	%	n	%	*p* *	OR (CI95%); *p* **
Year of admission					0.003	
2022	34	41%	7	14.6%		1.0
2023	24	28.9%	26	54.2%		2.59 (0.79–8.40); 0.11
2024	25	30.1%	15	31.2%		1.06 (0.27–4.26); 0.93
Age group					0.009	
<65 years	49	59%	17	35.4%		1.0
≥65 years	34	41%	31	64.6%		1.96 (0.79–4.84); 0.15
Marital status					0.006	
Single	14	16.9%	3	6.3%		1.0
Married	46	55.4%	17	35.4%		1.64 (0.36–7.37); 0.52
Widowed	11	13.3%	13	27.1%		2.73 (0.51–14.79); 0.24
Other	12	14.5%	15	31.2%		3.29 (0.61–17.68); 0.16
NYHA class					0.01	
II	23	27.7%	4	8.3%		1.0
III	31	37.4%	17	35.4%		2.11 (0.52–8.51); 0.29
IV	29	34.9%	27	56.3%		3.84 (0.89–16.55); 0.04
Anemia					0.02	
No	63	75.9%	27	56.3%		1.0
Yes	20	24.1%	21	43.8%		1.62 (0.66–3.96); 0.29
SGLT2i use					0.05	
No	59	71.1%	41	85.4%		1.0
Yes	24	28.9%	7	14.6%		0.50 (0.18–1.43); 0.20
Hospital readmissions					0.004	
No	69	83.1%	29	60.4%		1.0
Yes	14	16.9%	19	39.6%		2.15 (0.85–5.42); 0.11

* Chi-squared test; ** Logistic regression model (included all variables on the table). SGLT2i: Sodium-glucose cotransporter-2 inhibitors.

## Data Availability

The data that support the findings of this study are available as Supplementary Materials.

## Supplementary Materials

The following supporting information can be downloaded at: https://www.mdpi.com/article/10.3390/jcm14020644/s1.

## Abbreviations

The following abbreviations are used in this manuscript:

ACEI/ARB	Angiotensin-converting-enzyme inhibitors/angiotensin receptor blockers
ARNI	Angiotensin receptor-neprilysin inhibitors
BB	Beta-blockers
CAD	Coronary artery disease
CKD	Chronic kidney disease
CRT	Cardiac resynchronization therapy
HF	Heart failure
ICD	Implantable cardioverter defibrillator
LVEF	Left ventricle ejection fraction
MRA	Mineralocorticoid receptor antagonists
NYHA	New York Heart Association classification
QoC	Quality of care
SGLT2i	Sodium-glucose cotransporter-2 inhibitors
