# Supplementary material for: Evolution of the Quality of Care in Patients with Decompensated Heart Failure in a Venezuelan Hospital
Source: J Clin Med. 2025 Jan 20;14(2):644. doi: 10.3390/jcm14020644 (PMC11765786; doi:10.3390/jcm14020644)
Supplement: Supplementary file 1 [file jcm-14-00644-s001.zip › jcm-3417671-supplementary.pdf]

N°Case	Year	age	gender	Civil Status	Educational Status	Enployment
1	2023	88	M	MARRIED	BASIC	DESEMPLEAD
2	2023	60	F	MARRIED	BASIC	DESEMPLEAD
3	2023	59	F	OTHER	BASIC	DESEMPLEAD
4	2023	67	M	MARRIED	SUPERIOR	EMPLEADO
5	2023	73	F	WIDOW	SECONDARY	DESEMPLEAD
6	2023	90	F	WIDOW	ILLITERATE	DESEMPLEAD
7	2023	79	M	WIDOW	ILLITERATE	EMPLEADO
8	2023	69	M	OTHER	BASIC	EMPLEADO
9	2023	61	M	OTHER	SECONDARY	DESEMPLEAD
10	2023	63	F	OTHER	ILLITERATE	DESEMPLEAD
11	2023	90	M	WIDOW	BASIC	JUBILADO
12	2023	47	M	MARRIED	SECONDARY	EMPLEADO
13	2023	65	M	OTHER	BASIC	JUBILADO
14	2023	67	M	SINGLE	BASIC	JUBILADO
15	2023	45	F	SINGLE	SUPERIOR	DESEMPLEAD
16	2023	60	M	WIDOW	BASIC	DESEMPLEAD
17	2023	90	M	OTHER	ILLITERATE	DESEMPLEAD
18	2023	82	F	OTHER	ILLITERATE	DESEMPLEAD
19	2023	58	F	MARRIED	BASIC	DESEMPLEAD
20	2023	60	M	SINGLE	BASIC	JUBILADO
21	2023	86	F	SINGLE	BASIC	JUBILADO
22	2023	69	M	MARRIED	ILLITERATE	DESEMPLEAD
23	2023	47	M	MARRIED	BASIC	EMPLEADO
24	2023	47	M	MARRIED	SECONDARY	EMPLEADO
25	2023	60	F	OTHER	SECONDARY	EMPLEADO
26	2023	74	M	MARRIED	BASIC	DESEMPLEAD
27	2023	79	M	MARRIED	BASIC	JUBILADO
28	2023	52	M	SINGLE	SUPERIOR	DESEMPLEAD
29	2023	57	M	MARRIED	SUPERIOR	EMPLEADO
30	2023	68	F	OTHER	BASIC	DESEMPLEAD
31	2023	78	F	WIDOW	SECONDARY	DESEMPLEAD
32	2023	48	M	MARRIED	SECONDARY	DESEMPLEAD
33	2023	57	M	OTHER	SECONDARY	EMPLEADO
34	2023	65	F	WIDOW	BASIC	DESEMPLEAD
35	2023	80	F	OTHER	ILLITERATE	DESEMPLEAD
36	2023	53	M	MARRIED	SECONDARY	JUBILADO
37	2023	50	M	MARRIED	SECONDARY	EMPLEADO
38	2023	47	M	MARRIED	BASIC	DESEMPLEAD
39	2023	70	F	WIDOW	BASIC	DESEMPLEAD
40	2023	74	M	MARRIED	BASIC	DESEMPLEAD
41	2023	78	F	WIDOW	BASIC	DESEMPLEAD
42	2023	79	M	WIDOW	ILLITERATE	DESEMPLEAD
43	2023	63	F	MARRIED	BASIC	DESEMPLEAD
44	2023	57	M	WIDOW	SUPERIOR	DESEMPLEAD
45	2023	75	F	OTHER	BASIC	EMPLEADO
46	2023	45	M	MARRIED	BASIC	EMPLEADO
47	2023	77	M	MARRIED	BASIC	DESEMPLEAD
48	2023	62	M	MARRIED	SECONDARY	DESEMPLEAD
49	2023	44	F	MARRIED	SECONDARY	EMPLEADO

50	2023	63	F	MARRIED	SECONDARY	DESEMPLEAD
51	2024	34	M	SINGLE	BASIC	EMPLEADO
52	2024	83	F	WIDOW	SECONDARY	DESEMPLEAD
53	2024	60	M	MARRIED	SECONDARY	EMPLEADO
54	2024	73	F	OTHER	SECONDARY	DESEMPLEAD
55	2024	49	F	MARRIED	SECONDARY	EMPLEADO
56	2024	62	F	OTHER	SECONDARY	DESEMPLEAD
57	2024	71	F	OTHER	SECONDARY	DESEMPLEAD
58	2024	76	M	MARRIED	SECONDARY	DESEMPLEAD
59	2024	61	M	MARRIED	SUPERIOR	JUBILADO
60	2024	66	F	MARRIED	SUPERIOR	JUBILADO
61	2024	77	F	WIDOW	SUPERIOR	JUBILADO
62	2024	63	F	OTHER	SECONDARY	DESEMPLEAD
63	2024	64	F	MARRIED	SECONDARY	DESEMPLEAD
64	2024	47	M	MARRIED	SECONDARY	EMPLEADO
65	2024	43	M	OTHER	SUPERIOR	EMPLEADO
66	2024	83	F	WIDOW	SECONDARY	DESEMPLEAD
67	2024	72	F	OTHER	SUPERIOR	JUBILADO
68	2024	81	M	OTHER	ILLITERATE	DESEMPLEAD
69	2024	72	F	MARRIED	BASIC	DESEMPLEAD
70	2024	47	M	MARRIED	SECONDARY	EMPLEADO
71	2024	77	F	WIDOW	BASIC	DESEMPLEAD
72	2024	81	F	OTHER	BASIC	DESEMPLEAD
73	2024	87	F	OTHER	BASIC	DESEMPLEAD
74	2024	82	F	WIDOW	BASIC	DESEMPLEAD
75	2024	65	M	MARRIED	BASIC	DESEMPLEAD
76	2024	45	M	SINGLE	SECONDARY	DESEMPLEAD
77	2024	69	M	SINGLE	BASIC	DESEMPLEAD
78	2024	65	M	OTHER	SECONDARY	DESEMPLEAD
79	2024	59	F	MARRIED	BASIC	DESEMPLEAD
80	2024	40	M	OTHER	SECONDARY	EMPLEADO
81	2024	59	M	OTHER	ILLITERATE	DESEMPLEAD
82	2024	43	M	MARRIED	SECONDARY	EMPLEADO
83	2024	82	M	WIDOW	SECONDARY	DESEMPLEAD
84	2024	50	M	OTHER	SECONDARY	DESEMPLEAD
85	2024	66	M	OTHER	SECONDARY	DESEMPLEAD
86	2024	59	M	OTHER	SECONDARY	DESEMPLEAD
87	2024	75	M	SINGLE	ILLITERATE	DESEMPLEAD
88	2024	51	M	MARRIED	SECONDARY	EMPLEADO
89	2024	52	F	MARRIED	SECONDARY	EMPLEADO
90	2024	54	F	SINGLE	SECONDARY	DESEMPLEAD

Smoking Habit	Alcohol Intake	Regular PhyYEScal ac	HF Cause	NYHA Class
YES	YES	NO	ISCHEMIC	IV
NO	NO	NO	NO ISCHEMIC	IV
YES	NO	NO	ISCHEMIC	II
YES	YES	NO	ISCHEMIC	IV
NO	NO	NO	ISCHEMIC	III
NO	NO	NO	NO ISCHEMIC	IV
YES	YES	NO	ISCHEMIC	IV
YES	YES	NO	ISCHEMIC	III
YES	YES	NO	ISCHEMIC	II
YES	NO	NO	ISCHEMIC	IV
YES	YES	NO	ISCHEMIC	II
YES	YES	NO	ISCHEMIC	II
YES	YES	NO	ISCHEMIC	III
NO	YES	NO	ISCHEMIC	II
YES	YES	NO	NO ISCHEMIC	IV
YES	YES	NO	ISCHEMIC	IV
YES	NO	NO	ISCHEMIC	III
NO	NO	NO	NO ISCHEMIC	III
NO	YES	NO	ISCHEMIC	III
YES	YES	NO	ISCHEMIC	IV
YES	YES	NO	NO ISCHEMIC	IV
NO	NO	NO	ISCHEMIC	IV
NO	YES	NO	NO ISCHEMIC	IV
NO	NO	NO	NO ISCHEMIC	II
NO	NO	NO	ISCHEMIC	III
YES	YES	NO	ISCHEMIC	IV
NO	YES	NO	ISCHEMIC	IV
NO	NO	NO	NO ISCHEMIC	IV
YES	YES	NO	NO ISCHEMIC	IV
YES	NO	NO	ISCHEMIC	III
YES	NO	NO	ISCHEMIC	IV
YES	YES	NO	ISCHEMIC	IV
NO	YES	NO	NO ISCHEMIC	III
NO	NO	NO	ISCHEMIC	III
NO	YES	NO	ISCHEMIC	IV
YES	NO	NO	ISCHEMIC	III
NO	NO	NO	NO ISCHEMIC	III
NO	NO	NO	ISCHEMIC	III
NO	NO	NO	ISCHEMIC	III
NO	YES	NO	NO ISCHEMIC	IV
YES	NO	NO	NO ISCHEMIC	III
NO	NO	NO	ISCHEMIC	III
YES	YES	NO	ISCHEMIC	IV
YES	YES	NO	ISCHEMIC	IV
NO	NO	NO	ISCHEMIC	II
NO	NO	NO	NO ISCHEMIC	III
YES	YES	NO	NO ISCHEMIC	III
YES	YES	NO	ISCHEMIC	III
NO	YES	NO	ISCHEMIC	II

YES	NO	NO	ISCHEMIC	III
YES	YES	NO	DESCONOCIDA	IV
NO	NO	NO	NO ISCHEMIC	III
YES	YES	NO	ISCHEMIC	IV
NO	NO	NO	ISCHEMIC	IV
NO	NO	NO	NO ISCHEMIC	IV
NO	NO	NO	ISCHEMIC	IV
NO	NO	NO	NO ISCHEMIC	III
NO	YES	NO	DESCONOCIDA	IV
YES	YES	NO	ISCHEMIC	III
NO	YES	NO	DESCONOCIDA	IV
NO	NO	NO	NO ISCHEMIC	IV
NO	NO	NO	ISCHEMIC	III
NO	NO	NO	ISCHEMIC	IV
NO	YES	NO	NO ISCHEMIC	IV
NO	YES	NO	NO ISCHEMIC	IV
NO	NO	NO	NO ISCHEMIC	IV
NO	NO	NO	NO ISCHEMIC	IV
NO	YES	NO	ISCHEMIC	III
NO	NO	NO	ISCHEMIC	IV
NO	YES	NO	NO ISCHEMIC	IV
NO	NO	NO	ISCHEMIC	III
NO	NO	NO	ISCHEMIC	IV
NO	NO	NO	ISCHEMIC	IV
NO	NO	NO	ISCHEMIC	IV
NO	NO	NO	ISCHEMIC	IV
NO	YES	NO	ISCHEMIC	IV
NO	NO	NO	ISCHEMIC	III
NO	YES	NO	DESCONOCIDA	IV
NO	NO	NO	NO ISCHEMIC	III
NO	YES	NO	DESCONOCIDA	IV
YES	NO	NO	NO ISCHEMIC	III
YES	YES	NO	ISCHEMIC	IV
YES	YES	NO	NO ISCHEMIC	IV
NO	NO	NO	NO ISCHEMIC	IV
NO	YES	NO	NO ISCHEMIC	IV
YES	YES	NO	DESCONOCIDA	IV
YES	YES	NO	ISCHEMIC	III
NO	NO	NO	NO ISCHEMIC	IV
NO	NO	NO	NO ISCHEMIC	IV
NO	NO	NO	NO ISCHEMIC	III

N° Hospitalization in 12 months	Cons ACEI/ARB	Cons BB	Cons MRA
	NO	NO	NO
	NO	NO	NO
	YES	NO	NO
	NO	NO	NO
	NO	NO	YES
	YES	NO	NO
	NO	NO	NO
	NO	YES	YES
	NO	NO	NO
	NO	NO	NO
	YES	YES	YES
	NO	NO	NO
	YES	YES	NO
	YES	YES	YES
	NO	NO	NO
	YES	NO	NO
	YES	NO	NO
	YES	YES	NO
	NO	NO	NO
	YES	NO	NO
	YES	YES	NO
	YES	NO	NO
	YES	NO	NO
	YES	YES	YES
	YES	NO	NO
	NO	NO	NO
	YES	NO	NO
	NO	NO	NO
	YES	NO	NO
	NO	YES	NO
	YES	YES	YES
	YES	YES	YES
	YES	NO	YES
	YES	NO	NO
	NO	YES	YES
	NO	YES	YES
	NO	YES	YES
	NO	NO	NO
	YES	YES	YES
	YES	NO	NO
	YES	YES	NO
	NO	YES	YES
	NO	YES	YES
	NO	YES	YES
	YES	NO	NO
	YES	YES	YES
	NO	NO	NO
	NO	YES	NO
	NO	YES	YES

NO	YES	YES
NO	NO	NO
YES	YES	NO
NO	NO	NO
YES	YES	NO
NO	NO	NO
NO	YES	NO
NO	NO	YES
YES	YES	YES
NO	YES	YES
YES	YES	YES
NO	NO	NO
YES	NO	NO
YES	YES	NO
YES	NO	NO
YES	NO	YES
YES	NO	NO
YES	NO	NO
YES	NO	NO
YES	NO	NO
NO	NO	NO
YES	NO	NO
YES	YES	NO
YES	NO	NO
YES	YES	NO
YES	YES	NO
YES	YES	YES
NO	YES	YES
NO	NO	NO
NO	YES	NO
NO	YES	YES
NO	YES	NO
YES	YES	YES
YES	YES	NO
YES	NO	NO
NO	NO	NO
YES	NO	NO
NO	YES	YES
NO	NO	NO
NO	YES	NO
YES	NO	NO

Cons ARNI	Cons SGLT2i	Device	Hypertension	DM
NO	NO	NO	YES	NO
NO	NO	NO	NO	NO
NO	NO	NO	YES	YES
NO	NO	NO	YES	YES
NO	NO	NO	YES	NO
NO	NO	NO	YES	NO
NO	NO	NO	NO	YES
NO	NO	NO	YES	NO
NO	NO	NO	NO	NO
NO	NO	NO	NO	YES
NO	NO	NO	YES	NO
NO	NO	NO	NO	NO
NO	NO	NO	YES	YES
NO	NO	NO	YES	NO
NO	NO	NO	NO	NO
NO	NO	NO	YES	YES
NO	NO	NO	YES	NO
NO	NO	NO	YES	NO
NO	NO	NO	YES	NO
NO	NO	NO	YES	NO
NO	NO	NO	YES	YES
NO	NO	NO	YES	NO
NO	NO	NO	YES	NO
NO	YES	NO	YES	NO
NO	NO	NO	YES	YES
NO	NO	NO	YES	NO
NO	NO	NO	YES	NO
NO	YES	NO	NO	NO
NO	NO	NO	YES	NO
NO	NO	NO	YES	YES
NO	NO	NO	YES	NO
NO	NO	NO	YES	NO
NO	NO	NO	YES	YES
NO	NO	NO	YES	NO
YES	NO	NO	NO	NO
NO	NO	NO	YES	NO
NO	NO	NO	YES	NO
NO	NO	NO	YES	YES
NO	NO	NO	YES	YES
NO	NO	NO	YES	NO
NO	NO	NO	YES	YES
YES	YES	NO	YES	NO
YES	YES	NO	YES	NO
YES	YES	NO	YES	NO
NO	NO	NO	YES	YES
NO	YES	NO	YES	NO
NO	NO	NO	YES	NO
NO	NO	NO	YES	NO
YES	YES	NO	YES	NO

YES	YES	NO	YES	NO
NO	NO	NO	NO	NO
NO	NO	NO	NO	NO
NO	NO	NO	YES	NO
NO	NO	NO	YES	YES
NO	NO	NO	NO	YES
NO	YES	NO	YES	YES
NO	YES	NO	YES	YES
NO	YES	NO	NO	NO
YES	YES	YES	YES	NO
NO	NO	NO	NO	YES
NO	NO	NO	NO	NO
NO	NO	NO	YES	YES
NO	NO	NO	YES	NO
NO	NO	NO	YES	NO
NO	NO	NO	YES	NO
NO	NO	NO	YES	NO
NO	NO	NO	YES	NO
NO	NO	NO	YES	NO
NO	NO	NO	YES	YES
NO	NO	NO	NO	YES
NO	NO	NO	YES	NO
NO	YES	NO	YES	YES
NO	NO	NO	YES	NO
NO	NO	NO	YES	YES
NO	YES	NO	YES	YES
YES	YES	YES	YES	NO
NO	NO	NO	NO	NO
NO	NO	NO	NO	NO
NO	YES	NO	YES	NO
NO	NO	NO	YES	NO
NO	NO	NO	YES	NO
NO	YES	NO	YES	NO
NO	YES	NO	YES	NO
NO	NO	NO	YES	NO
NO	NO	NO	YES	YES
NO	YES	NO	NO	NO
NO	NO	NO	YES	NO
NO	NO	NO	YES	NO
NO	YES	NO	NO	NO
NO	NO	NO	YES	NO

Obesity	Anemia	high LDL	CAD	CKD	LVEF at hospitalization	BB at hospi
NO	YES	NM	YES	NO	NO	YES
NO	NO	NM	NO	NO	YES	YES
NO	NO	NM	YES	NO	NO	YES
NO	NO	NM	YES	NO	YES	YES
NO	NO	NM	YES	NO	NO	YES
NO	YES	NM	NO	YES	NO	YES
NO	NO	NM	YES	NO	NO	YES
NO	NO	NM	YES	NO	NO	YES
NO	NO	NM	YES	NO	YES	YES
NO	YES	NM	YES	NO	YES	NO
NO	YES	NM	YES	YES	YES	YES
NO	NO	NM	YES	YES	YES	YES
NO	YES	NM	YES	YES	NO	YES
NO	NO	NM	YES	NO	NO	NO
NO	NO	NM	NO	NO	NO	NO
NO	YES	NM	YES	YES	NO	YES
NO	NO	NM	YES	NO	NO	NO
NO	NO	NM	NO	NO	NO	YES
YES	NO	NM	YES	NO	YES	NO
NO	YES	NM	YES	YES	YES	NO
NO	YES	NM	NO	YES	NO	YES
NO	NO	NM	YES	NO	NO	YES
NO	YES	NM	NO	NO	YES	YES
NO	YES	NM	NO	YES	NO	YES
NO	YES	NM	YES	YES	NO	YES
NO	NO	NM	YES	NO	YES	YES
YES	NO	NM	YES	YES	NO	YES
NO	NO	NM	NO	NO	YES	YES
NO	NO	NM	NO	NO	NO	YES
NO	NO	NM	YES	NO	NO	NO
NO	NO	NM	YES	NO	NO	YES
NO	YES	NM	YES	NO	YES	YES
NO	NO	NM	NO	YES	YES	YES
YES	YES	NM	YES	YES	YES	YES
NO	YES	NM	YES	YES	NO	YES
YES	NO	NM	YES	NO	NO	NO
NO	YES	NM	NO	YES	YES	YES
NO	YES	NM	YES	NO	NO	YES
NO	YES	NM	YES	YES	YES	YES
NO	YES	NM	NO	YES	YES	YES
NO	NO	NM	NO	NO	NO	YES
NO	NO	NM	YES	NO	YES	YES
NO	NO	NM	YES	NO	YES	YES
NO	YES	NM	YES	YES	NO	YES
NO	NO	NM	YES	YES	NO	YES
NO	YES	NM	NO	YES	NO	NO
NO	YES	NM	NO	YES	NO	YES
NO	NO	NM	YES	NO	NO	YES
NO	NO	NM	YES	YES	YES	YES

NO	YES	NM	YES	YES	YES	YES
YES	NO	NM	NO	NO	YES	YES
NO	NO	NM	NO	NO	YES	YES
NO	YES	NM	YES	YES	YES	YES
NO	YES	NM	YES	YES	YES	YES
NO	NO	NM	NO	NO	YES	YES
YES	YES	NM	YES	YES	YES	YES
YES	YES	NM	NO	YES	YES	YES
NO	YES	NM	NO	NO	YES	YES
NO	YES	NM	YES	YES	YES	YES
NO	NO	NM	NO	NO	YES	YES
YES	NO	NM	NO	NO	YES	YES
NO	YES	NM	YES	YES	YES	NO
NO	YES	NM	YES	YES	YES	YES
YES	NO	NM	NO	NO	YES	YES
NO	NO	NM	NO	YES	YES	YES
NO	NO	NM	NO	YES	YES	YES
NO	YES	NM	NO	NO	YES	YES
NO	YES	NM	YES	YES	YES	YES
NO	YES	NM	YES	YES	YES	YES
NO	NO	NM	YES	YES	YES	NO
YES	NO	NM	YES	YES	YES	YES
NO	NO	NM	YES	NO	YES	YES
NO	YES	NM	YES	YES	YES	YES
NO	YES	NM	YES	YES	YES	YES
YES	NO	NM	YES	NO	YES	YES
YES	NO	NM	YES	NO	YES	YES
NO	NO	NM	YES	NO	YES	YES
NO	YES	NM	NO	YES	YES	YES
NO	NO	NM	NO	NO	YES	NO
YES	NO	NM	NO	NO	YES	YES
NO	NO	NM	NO	YES	YES	YES
YES	NO	NM	YES	NO	YES	YES
NO	NO	NM	NO	NO	YES	YES
YES	NO	NM	NO	YES	YES	YES
YES	NO	NM	NO	NO	YES	YES
NO	YES	NM	NO	YES	YES	YES
NO	NO	NM	YES	NO	YES	YES
NO	NO	NM	NO	NO	YES	YES
NO	NO	NM	NO	NO	YES	NO
YES	NO	NM	NO	NO	YES	YES

ACEI/ARB at hosp	Follow up progr	MRA at hosp
YES	YES	YES
NO	YES	YES
YES	YES	YES
YES	YES	YES
YES	YES	NO
YES	YES	YES
YES	YES	YES
YES	YES	YES
NO	YES	NO
NO	YES	NO
NO	YES	NO
YES	YES	NO
YES	YES	NO
YES	YES	YES
NO	YES	NO
YES	YES	YES
YES	YES	NO
YES	YES	YES
NO	YES	YES
NO	YES	NO
YES	YES	YES
YES	YES	YES
YES	YES	YES
YES	YES	YES
NO	YES	NO
NO	YES	YES
YES	YES	NO
YES	YES	YES
YES	NO	NO
YES	YES	NO
NO	YES	YES
YES	YES	YES
YES	YES	YES
YES	YES	YES
NO	YES	YES
YES	YES	YES
YES	YES	NO
YES	YES	YES
YES	YES	YES
YES	YES	NO
YES	YES	YES
YES	YES	YES
YES	YES	YES
YES	YES	YES
YES	YES	NO
YES	YES	NO
NO	YES	YES
YES	YES	NO
NO	YES	YES

YES	YES	NO
YES	YES	YES
YES	YES	NO
YES	YES	NO
YES	YES	NO
YES	YES	YES
YES	YES	NO
YES	YES	YES
YES	YES	YES
NO	YES	YES
NO	YES	NO
YES	YES	YES
YES	NO	NO
YES	YES	NO
YES	YES	YES
YES	YES	YES
YES	YES	YES
YES	YES	YES
YES	YES	YES
YES	YES	NO
YES	YES	NO
YES	YES	NO
NO	YES	NO
YES	YES	NO
YES	YES	YES
YES	YES	YES
YES	YES	YES
YES	YES	YES
NO	YES	YES
NO	YES	NO
YES	YES	YES
NO	YES	NO
YES	YES	YES
NO	YES	YES
YES	YES	YES
YES	YES	YES
YES	YES	NO
YES	YES	YES
YES	YES	YES
NO	YES	YES
YES	YES	YES

New Hospitalization at 6m	N° Hospitalizatio	Death 6m
YES	2	YES
YES	1	NO
NO		NO
NO		NO
NO		YES
NO		YES
YES	1	NO
NO		YES
YES	4	NO
NO		YES
NO		YES
NO		NO
YES	3	NO
NO		NO
NO		YES
NO		NO
NO		YES
YES	2	YES
NO		NO
NO		NO
NO		YES
YES	2	YES
NO		YES
NO		NO
YES	2	NO
NO		YES
NO		YES
YES	2	NO
NO		NO
NO		YES
YES	1	YES
NO		YES
YES	3	YES
NO		NO
NO		YES
NO		NO
NO		NO
NO		NO
NO		YES
NO		YES
YES	2	YES
YES	1	YES
NO		NO
NO		YES
YES	1	YES
NO		NO
NO		NO
YES	1	YES
NO		NO

NO		NO
NO		YES
NO		NO
YES	2	YES
NO		YES
NO		NO
NO		NO
YES	2	YES
YES	1	NO
NO		YES
NO		YES
NO		YES
NO		YES
NO		YES
NO		YES
YES	2	YES
YES	3	YES
NO		YES
NO		YES
YES	2	NO
NO		NO
YES	1	NO
YES	1	NO
NO		NO
NO		NO
NO		NO
NO		NO
NO		NO
YES	4	NO
NO		NO
NO		NO
NO		YES
NO		NO
NO		NO
NO		NO
NO		NO
NO		NO
NO		NO
YES	2	NO
NO		NO
NO		NO
